# Reversible Bilateral Fixed Dilated Pupils in Hypercapnic Respiratory Failure Following Chronic Graft Versus Host Disease: A Case Report and Review of Literature

**DOI:** 10.1002/ccr3.71201

**Published:** 2025-10-09

**Authors:** Aria Shirani, Ahmadreza Saghazadeh, Rasoul Aliannejad

**Affiliations:** ^1^ Shariati Hospital Tehran University of Medical Sciences Tehran Iran; ^2^ Division of Pulmonology and Critical Care, School of Medicine, Shariati Hospital Tehran University of Medical Sciences Tehran Iran

**Keywords:** autonomic neuropathy, graft versus host disease, hypercapnia, respiratory failure

## Abstract

The causes of bilateral fixed pupils in a patient with respiratory failure are diverse. Although rare, hypercapnia can also lead to this phenomenon, which is often reversible. This issue highlights the importance of a thorough evaluation of the patient's history, physical examination, laboratory data, medications, and imaging studies.

## 1 Background

Graft versus host disease (GVHD) is a multi‐system disorder that is a complication of blood product transfusion, solid organ transplant, or, more commonly, allogenic hematopoietic stem cell transplant (AHSCT) [1]. GVHD after an AHSCT is categorized into two types: acute and chronic, as acute GVHD occurs within the first three months following the transplant, while chronic GVHD is diagnosed when symptoms appear after three months [2]. Although there are some overlapping features between the two types, they also display distinct differences, such as chronic GVHD often resembling an autoimmune disorder and tending to affect the lungs more significantly than acute GVHD. Pulmonary GVHD primarily manifests as Bronchiolitis obliterans syndrome (BOS), characterized by new‐onset obstructive lung disease and air trapping. It causes progressive airway obstruction, which could lead to hypoventilation and respiratory failure [3, 4, 5].

The spectrum of skin involvement in GVHD is broad, ranging from erythematous maculopapular rashes to sclerosis, with the former being characteristic of chronic GVHD [2]. Additionally, neurological involvement has rarely been reported in patients with GVHD, but it can include immune‐mediated encephalitis, peripheral neuropathy, autonomic dysfunction, and neurocognitive deficits [6, 7].

There are a few case reports in the literature that discuss instances of concurrent respiratory failure and bilateral reversible fixed dilated pupils; however, these are limited to some specific neurologic disorders like Guillain–Barré syndrome and Myasthenia Gravis. To date, there have been no reports of this condition in patients with chronic GVHD [8, 9].

This report describes a 29‐year‐old male with a history of acute lymphoblastic leukemia (ALL) who underwent AHSCT six months after the diagnosis. Following the treatment, he developed chronic GVHD affecting his skin, lungs, and peripheral nervous system. He was admitted to the hospital with shortness of breath, generalized weakness, slightly reduced consciousness, and abdominal distension. Within two days of his admission, he experienced simultaneous bilateral fixed pupil dilation and hypercapnic respiratory failure.

## 2 Case History

A 29‐year‐old male with a history of hypothyroidism and Philadelphia‐like ALL at the age of 23 received an AHSCT from his human leukocyte antigen (HLA)‐matched sister six months after his diagnosis, following the completion of induction and consolidation regimens. One year after the transplant, the patient began experiencing skin involvement and exertional dyspnea. Further investigation revealed generalized skin sclerosis, particularly of the chest wall, and BOS, likely attributed to chronic GVHD. Consequently, treatment for chronic GVHD was initiated using immunosuppressive agents.

The patient was also diagnosed with major depressive disorder and subsequently received treatment for this condition. Despite years of therapy, the sclerosis of the chest wall skin led to restrictive lung disease, which, along with BOS, progressed over time. Additionally, the patient developed peripheral neuropathy, primarily presenting as distal paresthesia. Subsequently, by May 2024, he was reliant on home noninvasive ventilation (NIV) due to chronic hypercapnia. Six months later, following a severe episode of pneumonia, he was placed on invasive mechanical ventilation and was ultimately discharged with a tracheostomy tube, which he continued to depend on. Approximately one month before his current hospital admission, he suffered severe pneumonia and was treated with a regimen of antibiotics and antifungal medications. The medications the patient was using at home before current admission included olanzapine 2.5 mg daily plus escitalopram 10 mg daily, both of which were prescribed to treat underlying major depressive disorder, pirfenidone 800 mg twice daily, imatinib 100 mg twice daily, chlordiazepoxide 5 mg daily, levothyroxine 100 mcg daily, and prednisolone 5 mg twice daily.

## 3 Investigation

**FIGURE 1:**
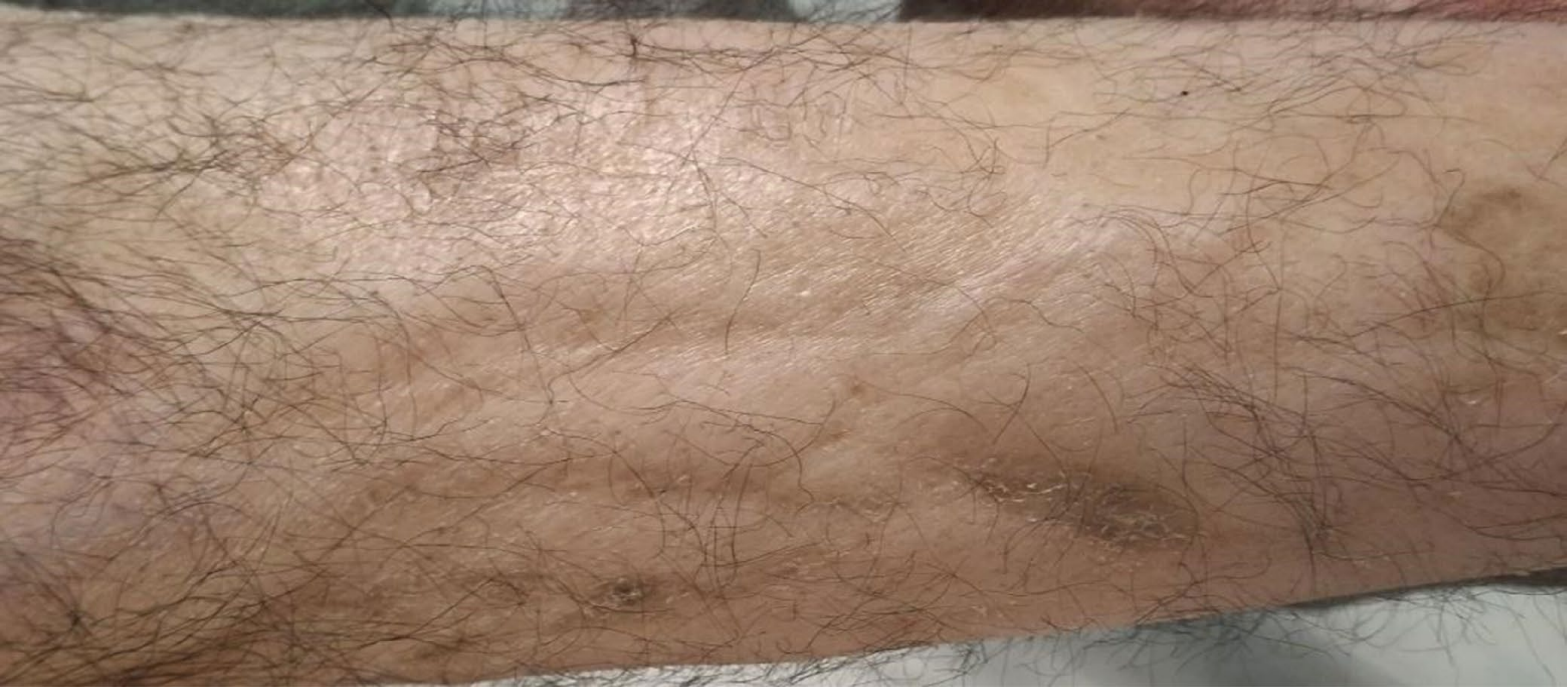
The skin of the patient indicating generalized sclerosis.

The patient presented to the Shariati Hospital Emergency Department with complaints of generalized weakness, mild decreased level of consciousness, and abdominal distension. The patient responded to verbal commands but was lethargic. His vital signs on admission indicated a heart rate of 118 bpm, blood pressure of 100/70 mmHg, respiratory rate of 27 bpm, and a saturation of peripheral oxygen (SpO_2_) in room air of 82%. On the physical examination, the patient's pupils were normal in size and reactive to light. The skin appeared generally sclerotic (Figure 1). Heart auscultation was unremarkable except for the presence of tachycardia. Lung auscultation showed bilateral diffuse crackles at the bases of both lungs. There was moderate abdominal distention, but no tenderness was noted upon palpation. The remainder of the examination revealed no pathological findings.

Laboratory studies revealed leukocytosis with a white blood cell count of 24,000 per microliter and no signs of anemia. Liver function tests indicated aspartate transaminase and alanine transaminase levels up to four times above the upper normal limit, while bilirubin levels were normal. Alkaline phosphatase was 1.5 times the upper limit, and an elevated C‐reactive protein (CRP) level of 131 mg/dL was noted. A venous blood gas (VBG) analysis showed respiratory acidosis with a pH of 7.27 and a pCO_2_ of 49.1 mmHg, and an HCO_3_ of 22.5 mEq/L.

**FIGURE 2:**
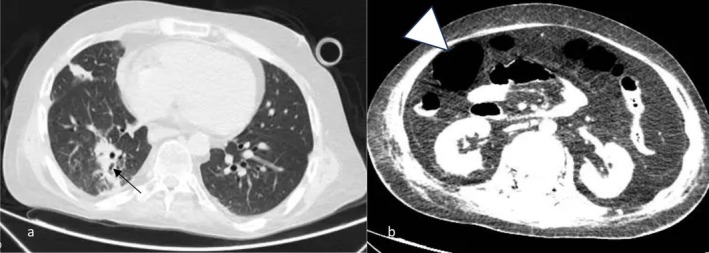
(a) Spiral chest computed tomography (CT) scan indicating bronchiolitis obliterans syndrome and right lower lobe consolidation (arrow), (b) Spiral abdomen‐pelvic CT scan showing dilated bowel loop (arrowhead) in favor of ileus.

Imaging studies, including a spiral chest computed tomography (CT) scan, indicated a mosaic attenuation pattern and consolidation in the right lower lobe, suggesting pneumonia (Figure 2a). A brain CT scan was unremarkable, and an abdominal‐pelvic scan revealed dilated colonic loops consistent with ileus (Figure 2b). The patient was admitted to the intensive care unit (ICU) and received intravenous antibiotics, gastrointestinal rest, and mechanical ventilation. The ventilation was set in assist‐control mode with volume control. The settings were as follows: Tidal Volume: 6 mL/kg of predicted body weight (PBW), fraction of inspired oxygen (FiO_2_): 0.6, positive end‐expiratory pressure (PEEP): 5 cm H_2_O, respiratory rate (RR): 20 breaths per minute, and plateau pressure: 25 cm H_2_O.

On the second day of admission, the patient experienced a gradual decrease in consciousness, accompanied by the sudden appearance of bilaterally dilated, nonreactive pupils. The carotid artery pulsations were palpable, and there were a few patient‐triggered breaths on the ventilator monitor, while blood pressure and heart rate remained stable. A non‐contrast brain CT ruled out intracranial hemorrhage, while a neurology consultation suspected an ischemic cerebrovascular accident (CVA). Urgent lab results showed severe respiratory failure with a VBG indicating a pH of 7.08, pCO_2_ of 81 mmHg, and HCO_3_ of 23.0 mEq/L; simultaneously, serum lactate level was 3.1 mmol/L.

The mechanical ventilation settings were adjusted by mild increases in tidal volume while preserving plateau pressure (below 30 cm H_2_O) and increasing RR, leading to a decrease in the patient's pCO_2_ level on VBG to 48 mmHg, accompanied by a pH of 7.28 and HCO_3_ of 22.7 mEq/L within 30 min. His pupils returned to normal size, and his level of consciousness improved. This suggests that the episode was likely caused by hypercapnic encephalopathy resulting from respiratory failure, rather than a primary brain injury, since the brain CT angiography was unremarkable, and the brain magnetic resonance imaging (MRI), with diffusion‐weighted sequences (DWI), showed no hyperintensities. Another episode of bilateral fixed nonreactive pupils occurred the following day due to hypercapnia, with VBG showing a pH of 7.03, pCO_2_ of 104 mmHg, and HCO_3_ of 27.4 mEq/L with a concurrent lactate level of 2.9 mmol/L. After adjusting the ventilator, the pCO_2_ on VBG declined to 50.6 mmHg with a pH of 7.23 and HCO_3_ of 21.4 mEq/L, and the pupils returned to normal size.

## 4 Follow‐Up and Outcome

The admission was complicated by acute kidney injury requiring dialysis, and deterioration of pneumonia ultimately resulted in the patient's death one month after admission.

## 5 Discussion

This case highlights a rare complication of hypercapnic respiratory failure in a 29‐year‐old man with a history of chronic GVHD. His symptoms were likely due to the deterioration of autonomic neuropathy caused by elevated carbon dioxide levels, which resulted in reversible bilateral fixed pupils and ileus. His condition could be linked to a neurological impairment, but a rapid response to respiratory correction confirmed this finding.

Respiratory failure can complicate the course of chronic GVHD when the lungs are involved. In our case, chest wall involvement exacerbated respiratory failure and hypercapnia. There are only a few case reports that describe hypercapnia‐induced mydriasis. For instance, Joyce et al. reported on a middle‐aged woman who experienced an asthma attack leading to hypercapnia, which subsequently caused brain edema. This brain edema resulted in a pressure effect that caused a unilateral, fixed, and dilated pupil. However, after correcting the carbon dioxide levels, the brain edema responded positively, and the pupil size returned to normal within the next 12 h [10]. In another report, a unilateral unresponsive pupil was observed in a 10‐year‐old child following an asthma attack, attributed to local contact with ipratropium in the affected eye. However, there was no evidence of ipratropium solution being instilled in the affected eye, and the explanation remained controversial [11].

Our patient experienced episodes of hypercapnia while on mechanical ventilation. These episodes could be attributed to the difficulties in ventilator settings, which were challenging due to chest wall restriction caused by skin sclerosis and obstructive airway disease resulting from BOS in the context of chronic GVHD [12].

The effects of hypercapnia and autonomic neuropathy on the pupil are distinct and complex. Autonomic neuropathy usually leads to more consistent pupillary changes. When both conditions occur together, the resulting pupillary signs likely reflect a combination of their effects. Typically, the signs will be primarily influenced by the underlying neuropathy, but they may also be affected by the severity of the hypercapnia [13]. Thus, it is assumed that CO_2_ facilitates reactions resulting in the production of reactive oxygen and nitrogen species, causing cellular membrane chemoreceptor dysfunction, as neural cell membranes are highly susceptible to oxidative stress, but this effect seems to be reversible [14]. An additional mechanism may involve sympathetic overactivity and parasympathetic dysfunction resulting from hypercapnia. The sensorimotor integration area of the limbic system, which perceives respiratory discomfort and regulates autonomic control, is connected to central sympathetic output in the brainstem [15, 16]. Both sympathetic overactivity and parasympathetic dysfunction respond rapidly to a decrease in CO_2_ concentration [15].

In our case, the primary diagnoses considered were cardiac arrest, brain death, hemorrhagic CVA with mass effect and cerebral herniation, brain edema due to hypercapnia, and, less likely, an ischemic stroke in the brain stem causing bilateral mydriasis, which is very rare and scarcely reported in the literature [17, 18]. We ruled out cardiac arrest and brain death by palpating the carotid artery pulsations and patient‐triggered ventilations, respectively. Moreover, based on the brain CT scan, we ruled out brain hemorrhage and edema, making the patient a candidate for thrombolytic therapy. However, after the patient's condition improved in response to respiratory correction and with an unremarkable brain CT angiography and MRI with DWI sequences, the thrombolytic therapy was canceled. This issue emphasizes the importance of making a differential diagnosis to avoid unnecessary and potentially harmful treatments.

We attributed the reversible fixed dilated pupils to autonomic neuropathy; however, there are also rare cases of neuropathy with bilateral pupils after microvascular decompression [19], Guillain‐Barré syndrome [20], and other autonomic neuropathies [21]. Furthermore, it is essential to consider medication‐induced mydriasis in every patient with respiratory failure in the ICU. The wide range of medications used in these patients, particularly sedatives and anticonvulsants, makes this consideration crucial, as documented in the literature [22, 23]. In our patient, none of the medications used at home or prescribed in the hospital were suspected of being involved in the event, making this diagnosis less likely.

In short, after ruling out cardiac arrest and brain death in this patient, we examined potential CNS issues such as brain edema and CVA. Upon finding no pathological abnormalities in the imaging studies, we conducted a thorough review of the patient's medications to ensure that no suspected agents were prescribed. Ultimately, we determined that the bilateral fixed dilated pupils were due to autonomic neuropathy exacerbated by hypercapnic encephalopathy. Additionally, the patient's improvement following respiratory correction and decline in CO_2_ level supported this assumption. Our literature review reveals no other cases of fixed dilated pupils associated with hypercapnic encephalopathy, making our case unique.

## 6 Conclusion

Chronic GVHD is a multi‐organ condition that commonly affects the lungs and skin. In rare cases, it can lead to peripheral and autonomic neuropathy. Lung involvement may result in respiratory failure and hypercapnia, which can worsen neuropathy and ultimately bilateral mydriasis. Fortunately, in our patient, this condition responded quickly to respiratory correction and reduction in CO_2_ level, emphasizing the importance of a thorough evaluation when considering the differential diagnosis of bilateral nonreactive dilated pupils.

## Author Contributions

**Aria Shirani:** conceptualization, data curation, investigation, resources, visualization, writing – original draft, writing – review and editing. **Ahmadreza Saghazadeh:** data curation, investigation, supervision, writing – original draft. **Rasoul Aliannejad:** conceptualization, project administration, supervision, writing – review and editing.

## Consent

Verbal and written consent was obtained from the patient for publishing this manuscript.

## Conflicts of Interest

The authors declare no conflicts of interest.

## Data Availability

The data supporting this study's findings can be obtained from the corresponding author upon reasonable request.
